# 4-Nitro-*N*′-[(1*E*,2*E*)-3-phenyl­prop-2-en-1-yl­idene]benzohydrazide

**DOI:** 10.1107/S1600536810011864

**Published:** 2010-04-02

**Authors:** Tanveer Ahmad, Muhammad Zia-ur-Rehman, Hamid Latif Siddiqui, Shahid Mahmud, Masood Parvez

**Affiliations:** a Institute of Chemistry, University of the Punjab, Lahore 54590, Pakistan; b Applied Chemistry Research Centre, PCSIR Laboratories Complex, Lahore 54600, Pakistan; c Department of Chemistry, The University of Calgary, 2500 University Drive NW, Calgary, Alberta, Canada T2N 1N4

## Abstract

In the title mol­ecule, C_16_H_13_N_3_O_3_, the benzene and phenyl rings are linked through a propenyl­idene hydrazide fragment, C—C(=O)—N(H)—N=C(H)—C(H)=C(H)—, which is fully extended with torsion angles in the range 175.4 (2)–179.9 (2)°. The dihedral angle between the the benzene and phenyl rings is 58.28 (7)°. In the crystal structure, inter­molecular N—H⋯O hydrogen bonds link the mol­ecules into chains along the *b* axis and additional stabilization is provided by weak inter­molecular C—H⋯O hydrogen bonds.

## Related literature

For the synthesis of related compounds, see: Ahmad *et al.* (2010 ▶); Küçükgüzel *et al.* (2007 ▶); Navidpour *et al.* (2006 ▶); Stocks *et al.* (2004 ▶). For the biological activity of benzohydrazides, see: Zia-ur-Rehman *et al.* (2009 ▶); Galal *et al.* (2009 ▶); Bordoloi *et al.* (2009 ▶). For a related structure, see: Ji & Shi (2008 ▶). For carbohydrazides, see: Rodríguez-Argüelles *et al.* (2004 ▶).
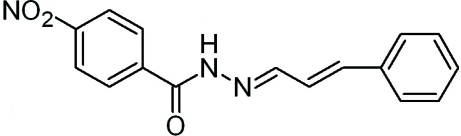


## Experimental

### 

#### Crystal data

C_16_H_13_N_3_O_3_
*M* *_r_* = 295.29
Monoclinic, 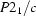

*a* = 16.4236 (17) Å
*b* = 5.3360 (5) Å
*c* = 17.1073 (18) Å
β = 114.578 (5)°
*V* = 1363.4 (2) Å^3^
*Z* = 4
Mo *K*α radiation
μ = 0.10 mm^−1^
*T* = 123 K
0.22 × 0.15 × 0.10 mm

#### Data collection

Nonius KappaCCD diffractometer
Absorption correction: multi-scan (*SORTAV*; Blessing, 1997 ▶) *T*_min_ = 0.978, *T*_max_ = 0.990
7965 measured reflections
2398 independent reflections
2194 reflections with *I* > 2σ(*I*)
*R*_int_ = 0.040

#### Refinement

*R*[*F* ^2^ > 2σ(*F* ^2^)] = 0.062
*wR*(*F* ^2^) = 0.130
*S* = 1.31
2398 reflections
199 parameters
H-atom parameters constrained
Δρ_max_ = 0.20 e Å^−3^
Δρ_min_ = −0.22 e Å^−3^

### 

Data collection: *COLLECT* (Hooft, 1998 ▶); cell refinement: *DENZO* (Otwinowski & Minor, 1997 ▶); data reduction: *SCALEPACK* (Otwinowski & Minor, 1997 ▶); program(s) used to solve structure: *SHELXS97* (Sheldrick, 2008 ▶); program(s) used to refine structure: *SHELXL97* (Sheldrick, 2008 ▶); molecular graphics: *ORTEP-3 for Windows* (Farrugia, 1997 ▶); software used to prepare material for publication: *SHELXL97*.

## Supplementary Material

Crystal structure: contains datablocks global, I. DOI: 10.1107/S1600536810011864/lh5023sup1.cif

Structure factors: contains datablocks I. DOI: 10.1107/S1600536810011864/lh5023Isup2.hkl

Additional supplementary materials:  crystallographic information; 3D view; checkCIF report

## Figures and Tables

**Table 1:** Hydrogen-bond geometry (Å, °)

*D*—H⋯*A*	*D*—H	H⋯*A*	*D*⋯*A*	*D*—H⋯*A*
N2—H2*N*⋯O3^i^	0.88	2.30	3.132 (3)	157
C8—H8⋯O3^i^	0.95	2.47	3.296 (3)	146
C14—H14⋯O1^ii^	0.95	2.56	3.305 (3)	135
C14—H14⋯O2^iii^	0.95	2.54	3.296 (3)	137

Symmetry codes: (i) 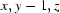; (ii) 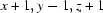; (iii) 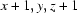.

**Fig. 1.**
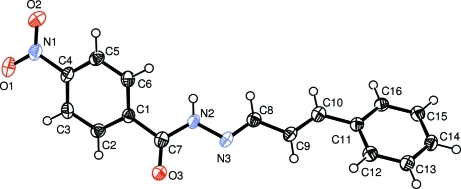
The title molecule with the displacement ellipsoids plotted at 50% probability level (Farrugia, 1997).

**Fig. 2.**
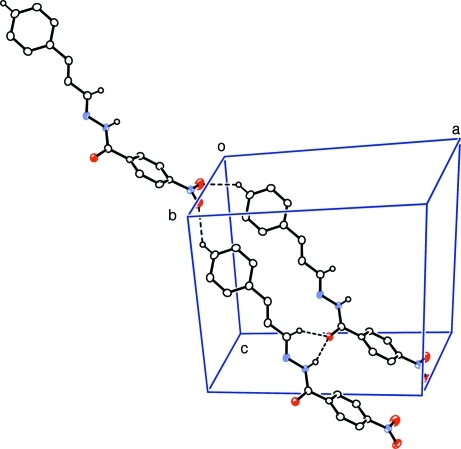
The unit cell packing of the title compound; H-bonds have been plotted with dashed lines and H-atoms not involved in H-bonds have been excluded for clarity.

## supplementary crystallographic information

### Comment

Hydrazides represent one of the most biologically active class of compounds, possessing a wide spectrum of activities such as anti-microbial (Zia-ur-Rehman *et al.*, 2009), anti-cancer (Galal *et al.*, 2009) and anti-genotoxic (Bordoloi *et al.*, 2009). These have been used as intermediates in the synthesis of oxadiazoles, triazoles and thiadiazoles (Küçükgüzel *et al.*, 2007; *et al.*, 2006; Stocks *et al*., 2004). Prompted by these observations and in continuation of our studies on the synthesis of various heterocyclic compounds (Ahmad *et al*., 2010; Zia-ur-Rehman *et al.*, 2009), we herein report the structure of the title compound (I).

In the the title compound (Fig. 1) the bond distances and angles agree with the corresponding bond distances and angles reported in a closely related compound (Ji & Shi, 2008). The benzene rings in (I) are linked through a propenylidenehydrazide fragment, C1/C7/N2/N3/C8/C9/C10, which is fully extended with torsion angles in the range 175.4 (2) and 179.9 (2)°. The dihedral angle between the two benzene rings is 58.28 (7)°. In the crystal structure, intermolecular N—H···O hydrogen bonds link the molecules into a chain along the *b*-axis and additional stabilization is provided by weak intermolecular C—H···O hydrogen bonds; details have been provided in Table. 1 and Fig. 2.

### Experimental

A mixture of para nitrobenzohydrazide (0.5 g, 2.76 mmoles), cinnamaldehyde (0.348 ml, 2.76 mmoles), orthophosphoric acid (0.2 ml) and methanol (50.0 ml) was refluxed for a period of 2 hours followed by removal of the solvent under vacuum. The contents were cooled and washed with cold methanol followed by crystallization from the same solvent at room temperature by slow evaporation. Yield: 94%. M.p. 516-517 K.

### Refinement

Though all the H atoms could be distinguished in the difference Fourier map the H-atoms were included at geometrically idealized positions and refined in riding-model approximation with N—H = 0.88 Å and C—H = 0.95 Å. The *U*_iso_(H) were allowed at 1.2*U*_eq_(N/C). The final difference map was essentially featurless.

### Crystal data

**Table:**

C_16_H_13_N_3_O_3_	*F*(000) = 616
*M**_r_* = 295.29	*D*_x_ = 1.439 Mg m^−^^3^
Monoclinic, *P*2_1_/*c*	Melting point: 516 K
Hall symbol: -P 2ybc	Mo *K*α radiation, λ = 0.71073 Å
*a* = 16.4236 (17) Å	Cell parameters from 5585 reflections
*b* = 5.3360 (5) Å	θ = 1.0–30.0°
*c* = 17.1073 (18) Å	µ = 0.10 mm^−^^1^
β = 114.578 (5)°	*T* = 123 K
*V* = 1363.4 (2) Å^3^	Prism, yellow
*Z* = 4	0.22 × 0.15 × 0.10 mm

### Data collection

**Table:**

Nonius KappaCCD diffractometer	2398 independent reflections
Radiation source: fine-focus sealed tube	2194 reflections with *I* > 2σ(*I*)
graphite	*R*_int_ = 0.040
ω and φ scans	θ_max_ = 25.0°, θ_min_ = 2.4°
Absorption correction: multi-scan (*SORTAV*; Blessing, 1997)	*h* = −19→19
*T*_min_ = 0.978, *T*_max_ = 0.990	*k* = −6→6
7965 measured reflections	*l* = −20→20

### Refinement

**Table:**

Refinement on *F*^2^	Primary atom site location: structure-invariant direct methods
Least-squares matrix: full	Secondary atom site location: difference Fourier map
*R*[*F*^2^ > 2σ(*F*^2^)] = 0.062	Hydrogen site location: difference Fourier map
*wR*(*F*^2^) = 0.130	H-atom parameters constrained
*S* = 1.31	*w* = 1/[σ^2^(*F*_o_^2^) + (0.0266*P*)^2^ + 1.5943*P*] where *P* = (*F*_o_^2^ + 2*F*_c_^2^)/3
2398 reflections	(Δ/σ)_max_ < 0.001
199 parameters	Δρ_max_ = 0.20 e Å^−^^3^
0 restraints	Δρ_min_ = −0.22 e Å^−^^3^

### Special details

**Table:**

Geometry. All esds (except the esd in the dihedral angle between two l.s. planes) are estimated using the full covariance matrix. The cell esds are taken into account individually in the estimation of esds in distances, angles and torsion angles; correlations between esds in cell parameters are only used when they are defined by crystal symmetry. An approximate (isotropic) treatment of cell esds is used for estimating esds involving l.s. planes.
Refinement. Refinement of *F*^2^ against ALL reflections. The weighted *R*-factor wR and goodness of fit *S* are based on *F*^2^, conventional *R*-factors *R* are based on *F*, with *F* set to zero for negative *F*^2^. The threshold expression of *F*^2^ > σ(*F*^2^) is used only for calculating *R*-factors(gt) etc. and is not relevant to the choice of reflections for refinement. *R*-factors based on *F*^2^ are statistically about twice as large as those based on *F*, and *R*- factors based on ALL data will be even larger.

### Fractional atomic coordinates and isotropic or equivalent isotropic displacement parameters (Å^2^)

**Table:**

	*x*	*y*	*z*	*U*_iso_*/*U*_eq_	
O1	0.03166 (12)	1.0652 (4)	0.43280 (12)	0.0336 (5)	
O2	0.08966 (14)	0.7047 (4)	0.42635 (14)	0.0444 (6)	
O3	0.42637 (12)	1.3627 (3)	0.78587 (11)	0.0251 (4)	
N1	0.09193 (15)	0.9101 (4)	0.45964 (14)	0.0282 (5)	
N2	0.44504 (13)	0.9434 (4)	0.81446 (13)	0.0208 (5)	
H2N	0.4231	0.7923	0.7981	0.025*	
N3	0.52157 (13)	0.9734 (4)	0.88984 (13)	0.0218 (5)	
C1	0.32370 (16)	1.0791 (5)	0.68390 (15)	0.0182 (5)	
C2	0.25118 (16)	1.2420 (5)	0.65552 (16)	0.0213 (6)	
H2	0.2538	1.3906	0.6871	0.026*	
C3	0.17520 (17)	1.1907 (5)	0.58186 (16)	0.0229 (6)	
H3	0.1255	1.3019	0.5622	0.028*	
C4	0.17367 (16)	0.9725 (5)	0.53757 (15)	0.0211 (6)	
C5	0.24504 (16)	0.8084 (5)	0.56339 (16)	0.0221 (6)	
H5	0.2423	0.6615	0.5310	0.026*	
C6	0.32073 (17)	0.8615 (5)	0.63727 (15)	0.0212 (6)	
H6	0.3705	0.7505	0.6562	0.025*	
C7	0.40351 (16)	1.1446 (5)	0.76550 (16)	0.0204 (6)	
C8	0.54992 (17)	0.7684 (5)	0.93255 (16)	0.0224 (6)	
H8	0.5171	0.6176	0.9125	0.027*	
C9	0.63111 (17)	0.7669 (5)	1.01056 (16)	0.0224 (6)	
H9	0.6635	0.9186	1.0302	0.027*	
C10	0.66201 (16)	0.5566 (5)	1.05602 (16)	0.0224 (6)	
H10	0.6255	0.4117	1.0365	0.027*	
C11	0.74637 (16)	0.5263 (5)	1.13289 (15)	0.0199 (5)	
C12	0.81544 (17)	0.7031 (5)	1.15687 (16)	0.0221 (6)	
H12	0.8087	0.8480	1.1225	0.027*	
C13	0.89376 (17)	0.6686 (5)	1.23042 (16)	0.0242 (6)	
H13	0.9399	0.7910	1.2464	0.029*	
C14	0.90515 (16)	0.4560 (5)	1.28093 (16)	0.0235 (6)	
H14	0.9586	0.4336	1.3315	0.028*	
C15	0.83804 (16)	0.2771 (5)	1.25705 (16)	0.0223 (6)	
H15	0.8458	0.1306	1.2909	0.027*	
C16	0.75935 (16)	0.3118 (5)	1.18347 (15)	0.0199 (5)	
H16	0.7138	0.1877	1.1674	0.024*	

### Atomic displacement parameters (Å^2^)

**Table:**

	*U*^11^	*U*^22^	*U*^33^	*U*^12^	*U*^13^	*U*^23^
O1	0.0222 (10)	0.0422 (12)	0.0276 (10)	0.0033 (9)	0.0016 (8)	0.0063 (9)
O2	0.0420 (13)	0.0311 (12)	0.0397 (12)	−0.0064 (10)	−0.0033 (10)	−0.0095 (10)
O3	0.0251 (10)	0.0190 (10)	0.0251 (10)	−0.0038 (8)	0.0044 (8)	−0.0010 (8)
N1	0.0250 (12)	0.0297 (13)	0.0236 (12)	−0.0064 (11)	0.0038 (10)	0.0034 (10)
N2	0.0172 (10)	0.0185 (11)	0.0199 (11)	−0.0029 (9)	0.0009 (9)	0.0007 (9)
N3	0.0175 (11)	0.0223 (11)	0.0202 (11)	−0.0023 (9)	0.0025 (9)	−0.0020 (9)
C1	0.0183 (12)	0.0175 (12)	0.0183 (12)	−0.0011 (10)	0.0073 (10)	0.0037 (10)
C2	0.0233 (13)	0.0162 (12)	0.0232 (13)	0.0001 (10)	0.0084 (11)	−0.0009 (10)
C3	0.0197 (13)	0.0225 (13)	0.0238 (13)	0.0045 (11)	0.0062 (11)	0.0050 (11)
C4	0.0194 (13)	0.0230 (13)	0.0177 (12)	−0.0033 (11)	0.0045 (10)	0.0041 (10)
C5	0.0245 (14)	0.0199 (13)	0.0204 (13)	−0.0014 (11)	0.0081 (11)	−0.0008 (10)
C6	0.0215 (13)	0.0188 (13)	0.0217 (13)	0.0025 (10)	0.0074 (11)	0.0024 (10)
C7	0.0189 (13)	0.0201 (13)	0.0229 (13)	−0.0010 (10)	0.0093 (11)	−0.0003 (10)
C8	0.0221 (13)	0.0199 (13)	0.0248 (13)	0.0001 (11)	0.0095 (11)	−0.0010 (11)
C9	0.0202 (13)	0.0211 (13)	0.0214 (13)	−0.0027 (11)	0.0041 (10)	−0.0040 (11)
C10	0.0198 (13)	0.0213 (13)	0.0255 (13)	−0.0020 (11)	0.0088 (11)	−0.0034 (11)
C11	0.0190 (13)	0.0211 (13)	0.0199 (12)	0.0013 (10)	0.0083 (10)	−0.0027 (10)
C12	0.0252 (14)	0.0165 (13)	0.0250 (13)	0.0014 (11)	0.0108 (11)	0.0008 (11)
C13	0.0195 (13)	0.0235 (13)	0.0275 (14)	−0.0013 (11)	0.0077 (11)	−0.0021 (11)
C14	0.0182 (13)	0.0265 (14)	0.0217 (13)	0.0052 (11)	0.0043 (10)	−0.0007 (11)
C15	0.0245 (13)	0.0201 (13)	0.0218 (13)	0.0037 (11)	0.0089 (11)	0.0020 (10)
C16	0.0222 (13)	0.0160 (12)	0.0221 (13)	−0.0010 (10)	0.0098 (11)	−0.0016 (10)

### Geometric parameters (Å, °)

**Table:**

O1—N1	1.224 (3)	C6—H6	0.9500
O2—N1	1.228 (3)	C8—C9	1.442 (3)
O3—C7	1.228 (3)	C8—H8	0.9500
N1—C4	1.484 (3)	C9—C10	1.339 (4)
N2—C7	1.358 (3)	C9—H9	0.9500
N2—N3	1.386 (3)	C10—C11	1.470 (3)
N2—H2N	0.8800	C10—H10	0.9500
N3—C8	1.290 (3)	C11—C16	1.397 (3)
C1—C2	1.389 (3)	C11—C12	1.399 (4)
C1—C6	1.398 (3)	C12—C13	1.388 (4)
C1—C7	1.506 (3)	C12—H12	0.9500
C2—C3	1.383 (4)	C13—C14	1.391 (4)
C2—H2	0.9500	C13—H13	0.9500
C3—C4	1.384 (4)	C14—C15	1.385 (4)
C3—H3	0.9500	C14—H14	0.9500
C4—C5	1.380 (4)	C15—C16	1.391 (3)
C5—C6	1.384 (3)	C15—H15	0.9500
C5—H5	0.9500	C16—H16	0.9500
			
O1—N1—O2	124.5 (2)	N3—C8—C9	120.4 (2)
O1—N1—C4	118.3 (2)	N3—C8—H8	119.8
O2—N1—C4	117.3 (2)	C9—C8—H8	119.8
C7—N2—N3	120.8 (2)	C10—C9—C8	121.6 (2)
C7—N2—H2N	119.6	C10—C9—H9	119.2
N3—N2—H2N	119.6	C8—C9—H9	119.2
C8—N3—N2	113.8 (2)	C9—C10—C11	126.9 (2)
C2—C1—C6	119.9 (2)	C9—C10—H10	116.5
C2—C1—C7	117.9 (2)	C11—C10—H10	116.5
C6—C1—C7	122.2 (2)	C16—C11—C12	118.3 (2)
C3—C2—C1	120.8 (2)	C16—C11—C10	119.3 (2)
C3—C2—H2	119.6	C12—C11—C10	122.4 (2)
C1—C2—H2	119.6	C13—C12—C11	120.6 (2)
C2—C3—C4	118.0 (2)	C13—C12—H12	119.7
C2—C3—H3	121.0	C11—C12—H12	119.7
C4—C3—H3	121.0	C12—C13—C14	120.4 (2)
C5—C4—C3	122.6 (2)	C12—C13—H13	119.8
C5—C4—N1	118.4 (2)	C14—C13—H13	119.8
C3—C4—N1	119.0 (2)	C15—C14—C13	119.6 (2)
C4—C5—C6	118.9 (2)	C15—C14—H14	120.2
C4—C5—H5	120.6	C13—C14—H14	120.2
C6—C5—H5	120.6	C14—C15—C16	120.1 (2)
C5—C6—C1	119.7 (2)	C14—C15—H15	120.0
C5—C6—H6	120.1	C16—C15—H15	120.0
C1—C6—H6	120.1	C15—C16—C11	120.9 (2)
O3—C7—N2	123.9 (2)	C15—C16—H16	119.5
O3—C7—C1	121.9 (2)	C11—C16—H16	119.5
N2—C7—C1	114.1 (2)		
			
C7—N2—N3—C8	175.4 (2)	C2—C1—C7—O3	−33.6 (4)
C6—C1—C2—C3	0.7 (4)	C6—C1—C7—O3	146.9 (3)
C7—C1—C2—C3	−178.9 (2)	C2—C1—C7—N2	144.5 (2)
C1—C2—C3—C4	0.1 (4)	C6—C1—C7—N2	−35.0 (3)
C2—C3—C4—C5	−1.0 (4)	N2—N3—C8—C9	177.3 (2)
C2—C3—C4—N1	178.4 (2)	N3—C8—C9—C10	−179.9 (2)
O1—N1—C4—C5	−174.1 (2)	C8—C9—C10—C11	175.6 (2)
O2—N1—C4—C5	5.8 (3)	C9—C10—C11—C16	165.8 (3)
O1—N1—C4—C3	6.5 (3)	C9—C10—C11—C12	−15.3 (4)
O2—N1—C4—C3	−173.6 (2)	C16—C11—C12—C13	−1.8 (4)
C3—C4—C5—C6	1.1 (4)	C10—C11—C12—C13	179.4 (2)
N1—C4—C5—C6	−178.3 (2)	C11—C12—C13—C14	0.7 (4)
C4—C5—C6—C1	−0.3 (4)	C12—C13—C14—C15	0.5 (4)
C2—C1—C6—C5	−0.6 (4)	C13—C14—C15—C16	−0.7 (4)
C7—C1—C6—C5	178.9 (2)	C14—C15—C16—C11	−0.3 (4)
N3—N2—C7—O3	−4.4 (4)	C12—C11—C16—C15	1.5 (4)
N3—N2—C7—C1	177.5 (2)	C10—C11—C16—C15	−179.6 (2)

### Hydrogen-bond geometry (Å, °)

**Table:**

*D*—H···*A*	*D*—H	H···*A*	*D*···*A*	*D*—H···*A*
N2—H2N···O3^i^	0.88	2.30	3.132 (3)	157
C8—H8···O3^i^	0.95	2.47	3.296 (3)	146
C14—H14···O1^ii^	0.95	2.56	3.305 (3)	135
C14—H14···O2^iii^	0.95	2.54	3.296 (3)	137

Symmetry codes: (i) *x*, *y*−1, *z*; (ii) *x*+1, *y*−1, *z*+1; (iii) *x*+1, *y*, *z*+1.
